# A new approach to identifying patients with elevated risk for Fabry disease using a machine learning algorithm

**DOI:** 10.1186/s13023-021-02150-3

**Published:** 2021-12-20

**Authors:** John L. Jefferies, Alison K. Spencer, Heather A. Lau, Matthew W. Nelson, Joseph D. Giuliano, Joseph W. Zabinski, Costas Boussios, Gary Curhan, Richard E. Gliklich, David G. Warnock

**Affiliations:** 1 Division of Cardiovascular Diseases, University of Tennessee Health Science Center, Memphis, TN USA; 2 OM1, Boston, MA USA; 3 Division of Neurogenetics, Department of Neurology, New York University, New York, NY USA; 4 Amicus Therapeutics, Inc., Cranbury, NJ USA; 5 Department of Medicine, Division of Nephrology, University of Alabama at Birmingham, Birmingham, AL USA

**Keywords:** Fabry disease, AI, Patient identification, Phenotypic biomarker

## Abstract

**Background:**

Fabry disease (FD) is a rare genetic disorder characterized by glycosphingolipid accumulation and progressive damage across multiple organ systems. Due to its heterogeneous presentation, the condition is likely significantly underdiagnosed. Several approaches, including provider education efforts and newborn screening, have attempted to address underdiagnosis of FD across the age spectrum, with limited success. Artificial intelligence (AI) methods present another option for improving diagnosis. These methods isolate common health history patterns among patients using longitudinal real-world data, and can be particularly useful when patients experience nonspecific, heterogeneous symptoms over time. In this study, the performance of an AI tool in identifying patients with FD was analyzed. The tool was calibrated using de-identified health record data from a large cohort of nearly 5000 FD patients, and extracted phenotypic patterns from these records. The tool then used this FD pattern information to make individual-level estimates of FD in a testing dataset. Patterns were reviewed and confirmed with medical experts.

**Results:**

The AI tool demonstrated strong analytic performance in identifying FD patients. In out-of-sample testing, it achieved an area under the receiver operating characteristic curve (AUROC) of 0.82. Strong performance was maintained when testing on male-only and female-only cohorts, with AUROCs of 0.83 and 0.82 respectively. The tool identified small segments of the population with greatly increased prevalence of FD: in the 1% of the population identified by the tool as at highest risk, FD was 23.9 times more prevalent than in the population overall. The AI algorithm used hundreds of phenotypic signals to make predictions and included both familiar symptoms associated with FD (e.g. renal manifestations) as well as less well-studied characteristics.

**Conclusions:**

The AI tool analyzed in this study performed very well in identifying Fabry disease patients using structured medical history data. Performance was maintained in all-male and all-female cohorts, and the phenotypic manifestations of FD highlighted by the tool were reviewed and confirmed by clinical experts in the condition. The platform’s analytic performance, transparency, and ability to generate predictions based on existing real-world health data may allow it to contribute to reducing persistent underdiagnosis of Fabry disease.

## Background

Fabry disease is an inherited X-linked disorder caused by mutations in the GLA gene that result in deficient or absent lysosomal α-Gal A activity, and intracellular accumulation of globotriaosylceramide (Gb3) and related glycosphingolipids [1–3]. The condition is progressive, due to the cumulative damage done to multiple organ systems, especially the heart, kidney and central and peripheral nervous systems [4]. Fabry disease varies substantially in age of onset and clinical presentation. ‘Classic’ Fabry disease is associated with onset in younger males who first experience neuropathic pain and gastrointestinal symptoms, including abdominal pain and diarrhea, and renal function deterioration. Patients also experience cardiac signs and symptoms (including arrhythmias, myocardial fibrosis, and left ventricular hypertrophy), and frequently suffer strokes and transient ischemic attacks. Later onset Fabry disease affects male and female patients. The presentation can be heterogenous including a range of symptoms including cardiac, central nervous system (CNS) and renal involvement, mood disorders, hearing loss, neuropathic pain and gastrointestinal (GI) symptoms [5]. Later onset symptoms vary in severity, and progress at different rates. Across the spectrum of Fabry disease, cardiac disease accounts for the majority of deaths in Fabry disease patients [6]. Although Fabry disease was once thought to exclusively affect males, both male and female patients may experience severe clinical manifestations [7].

Estimates of Fabry disease prevalence vary substantially, ranging in occurrence from 1 in 40,000 to 1 in 117,000 live births worldwide [8]. However, due to the variations in multisystemic clinical manifestations, Fabry disease remains substantially underdiagnosed [9]. The challenge of reaching a diagnosis for the full range of patients is made more difficult by a general lack of awareness among clinicians of the true variability in Fabry disease presentation [10]. Definitive diagnosis is achieved only through measured deficiency in ɑ-galactosidase A in males or by detection of a pathogenic *GLA* mutation [10]. The multisystemic nature of the disease’s manifestations compounded by lack of awareness has led to an unmet need as patients often experience significant delays between symptom onset and diagnosis, with symptoms frequently first occurring in childhood or early adolescence but formal diagnosis frequently reached only in patients’ 20’s or 30’s [11].

Prior efforts to improve rates of diagnosis of Fabry disease and to shorten time-to-diagnosis have been made. Education among healthcare providers to improve familiarity with and awareness of symptoms and disease trajectory has been expanded, with some success in increasing screening and eventual diagnosis rates [12]. Newborn screening for Fabry disease has been implemented in Taiwan, and some US states. Screening programs have detected *GLA* mutations at much higher rates than current estimates of Fabry disease prevalence in the general population, though not all mutations necessarily result in development of clinically significant Fabry disease [13, 14]. Finally, patient identification through screening of patients with certain conditions associated with Fabry disease (e.g., hypertrophic cardiomyopathy, renal failure) has been attempted [15, 16]. While frequently effective in identifying patients, this method relies on symptoms that only become evident once the disease has progressed substantially. In all efforts to diagnose Fabry disease patients, identifying the first case of the condition within a family is crucial. Other close relatives of the index case patient may be at much greater likelihood of having the disease, and can be evaluated accordingly.

Artificial intelligence (AI) provides a different approach for patient identification. AI methods isolate statistical patterns in large datasets and have been successfully used to predict patient outcomes in clinical settings [17]. These methods can function as ‘general [electronic health record] pattern recognition experts’ [18] and are especially useful in analyzing highly heterogeneous diseases where patients exhibit wide ranges of symptoms and clinical findings over long periods of time. Using AI methods for disease identification relies on assessing the presence of phenotypic patterns in the medical history of an undiagnosed patient to estimate likelihood of disease. These patterns can be learned from known Fabry disease patients’ medical histories. Though clinical applications of this technology are still nascent, AI methods have demonstrated strong performance in patient identification across a range of fields, including ophthalmology, neurology, cardiology, gastroenterology, and hepatology, among others [19–22].

Here, we describe an AI tool used to identify patients with Fabry disease. The tool (OM1 Patient Finder™, OM1 Inc., Boston, MA) was examined to determine its ability to identify Fabry disease cases using health history data. In addition to assessing statistical performance, including in men and women separately, the tool’s use of different features in healthcare records was studied to evaluate correspondence with known clinical signs and symptoms of the disease.

## Methods

The data used in this study were drawn from a large cloud-based curated dataset (the OM1 Real World Data Cloud, OM1, Inc, Boston, MA). This dataset is derived from deterministically linked, de-identified, patient-level health care claims, EMR, and other data, and includes medication history and prescription information, laboratory results, symptoms and signs, procedures, and diagnoses. Additional medical and pharmacy claims data are linked to these clinical data to provide further information regarding patients’ clinical care. These data cover January 1, 2013 to the present day, and represent patients with a wide age and geographic distribution (including patients from all 50 U.S. states). Use of these de-identified data to study patient characteristics and outcomes in retrospective, non-interventional, secondary analyses has been determined to be exempt from institutional review board (IRB) oversight by an independent IRB.

The dataset used in this study contained 4978 patients with confirmed Fabry disease, and 1,000,000 patients without any diagnostic or medication codes that would indicate a Fabry disease diagnosis. The patients with confirmed Fabry disease were identified by the presence of at least one Fabry disease ICD-10 code (E75.21, Fabry (-Anderson) disease), or by evidence of a medication approved to treat Fabry disease (agalsidase beta / Fabrazyme, or migalastat / Galafold) in their medical record. Patients without any of these indicators of a Fabry disease diagnosis were randomly selected from a population of several million patients with evidence of a minimum amount of activity in their medical records. Records were restricted to the period from January 1, 2013 to July 1, 2020. All patients were at least 18 years old for the entirety of the study period.

Following predictive AI modeling methodology [23], the study dataset (including patients with and without confirmed Fabry disease) was divided into two cohorts: a ‘training’ cohort comprising 75% of all patients selected at random, and a ‘testing’ cohort comprising the remaining 25%. The AI algorithm was calibrated to estimate the presence of Fabry disease using records from the training cohort.

The AI tool first assessed patients by computing a personal phenotypic signature for each patient, using longitudinal health history data. This signature comprised a collection of related phenotypic characteristics (e.g. reports of symptoms, use of medications, records of procedures), grouped together, and reviewed and labeled with clinically descriptive signifiers by authors with medical expertise. For example, a phenotypic signal labeled ‘neuropathy’ contained diagnosis codes for polyneuropathy and skin paresthesia, and procedure codes indicating nerve conduction studies and needle electromyography, as well as many others. Most relevant signals calculated by the AI tool corresponded to organ systems and categories of pathologies. The statistical strength and relevance of a signal for each individual patient’s predicted likelihood of undiagnosed Fabry disease is dependent on that signal’s manifestation in that patient's history.

**Fig. 1:**
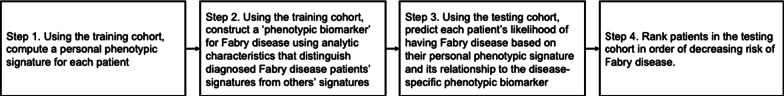
Flow diagram illustrating the tool’s process in assessing patient-level risk of Fabry disease

The tool then learned analytic relationships between patients’ phenotypic signatures and the outcome of interest—here, diagnosed Fabry disease—using the confirmed Fabry disease and non-confirmed patient data cohorts. Differences between the cohorts were used to construct a statistical ‘phenotypic biomarker’ for Fabry disease. As a final step, the platform predicted a likelihood of having Fabry disease for each patient in the testing cohort based on that patient’s phenotypic signature profile and its relationship to the phenotypic biomarker. Analytic performance in this classification task was quantified using receiver operating characteristic (ROC) curve analytic process represented in Fig. 1.

In addition, predictive performance was studied by examining effective Fabry disease ‘prevalence’ in groups of patients identified by the algorithm as at highest risk of having Fabry disease following stratification by risk. The tool rank-ordered patients in the testing cohort from greatest to least predicted Fabry disease likelihood and counted the number of confirmed Fabry disease patients in different risk strata (e.g., within the 1% of patients at greatest risk by predicted likelihood). Dividing these counts by the total number of patients in a risk group yielded an effective ‘prevalence’ for that risk group.

The study dataset was highly enriched with confirmed Fabry disease patients, containing approximately 1 confirmed patient for every 50 patients without confirmed Fabry disease (roughly three orders of magnitude greater than expected background prevalence). As such, calculated ‘prevalence’ values in risk groups were much higher than expected background prevalence of Fabry disease in the general population. To normalize, projected prevalence within these higher-risk strata was extrapolated using a conservative baseline population-wide assumption of 1 in 50,000 based on existing prevalence estimates [8]. For example, if calculated prevalence in a higher-risk stratum was 1 in 10 (that is, five times greater than the study population overall), projected ‘real-world’ prevalence after correcting for study population enrichment would be five times greater than the 1 in 50,000 background assumption, or 1 in 10,000.

The algorithm’s performance was also assessed by examining relative presence of phenotypic signals in different higher-risk strata. Within each, the fraction of patients with a particular phenotypic signal in their health history was calculated, and these frequencies of occurrence were compared across risk strata to identify patterns in phenotypic signal presentation in higher- and lower-risk groups.

Finally, these analyses were repeated after dividing the testing cohort into male-only and female-only subcohorts to examine robustness of predictive power while stratifying by sex.

Following generation and assessment of the tool’s analytic performance, a group of Fabry disease experts was assembled to review outputs, including components from patients’ individual phenotypic signatures and the platform’s phenotypic biomarker for Fabry disease. This review was intended to establish concordance between medical understanding of the condition and the algorithm’s outputs across results, especially considering the phenotypic variety in disease presentation in patients’ medical records.

## Results

The study population comprised a total of 1,004,978 patients. Relevant summary characteristics of the population are presented in Table 1.

**Table 1:** Age and sex distribution of study population

	Patients with confirmed Fabry disease (n = 4978)	Patients with no confirmed Fabry disease diagnosis (n = 1,000,000)
Sex (% female)	54.5%	55.1%
Mean age (SD)	47.0 (21.8)	44.8 (24.6)

**Fig. 2:**
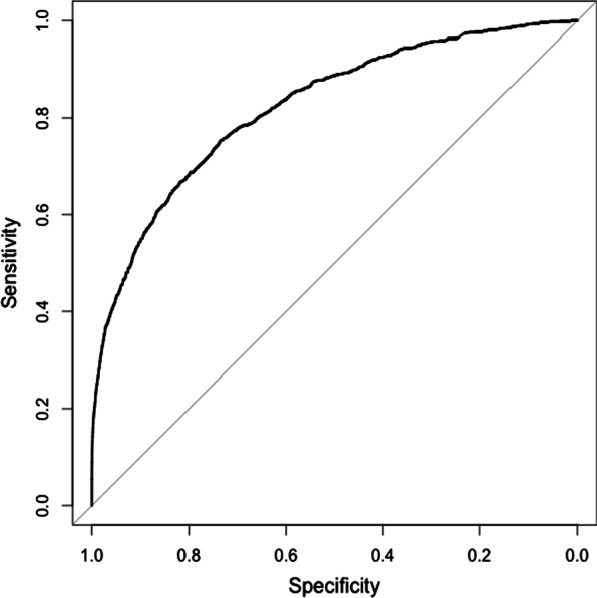
Receiver operating characteristic (ROC) curve. Area under the curve (AUC): 0.82

The tool demonstrated very strong analytic performance in identifying Fabry disease patients in the test cohort, with an overall area under the receiver operating curve (AUROC) of 0.82 (Fig. 2).

Following rank-ordering of test set patients by predicted risk of Fabry disease, the tool performed very well in concentrating Fabry disease patients at the riskier end of this ranking. Confirmed Fabry disease patient presence in the riskiest 1% of patients as identified by the algorithm was nearly 24-fold greater than the baseline prevalence level. Using a true population assumption of Fabry disease prevalence of 1 in 50,000, simulated prevalence in the 1% identified by the platform would be roughly 24 times greater, or 1 in 2100. Additional amplifications and projected prevalence calculations are provided in Table 2.

**Table 2:** Amplification in riskier strata of the testing set, following rank-ordering by predicted likelihood of Fabry disease

Risk group	Amplification relative to total population	Projected prevalence
Riskiest 10%	5.4×	1 in 9261
Riskiest 1%	23.9×	1 in 2090
Riskiest 0.1%	109.8×	1 in 455

**Fig. 3:**
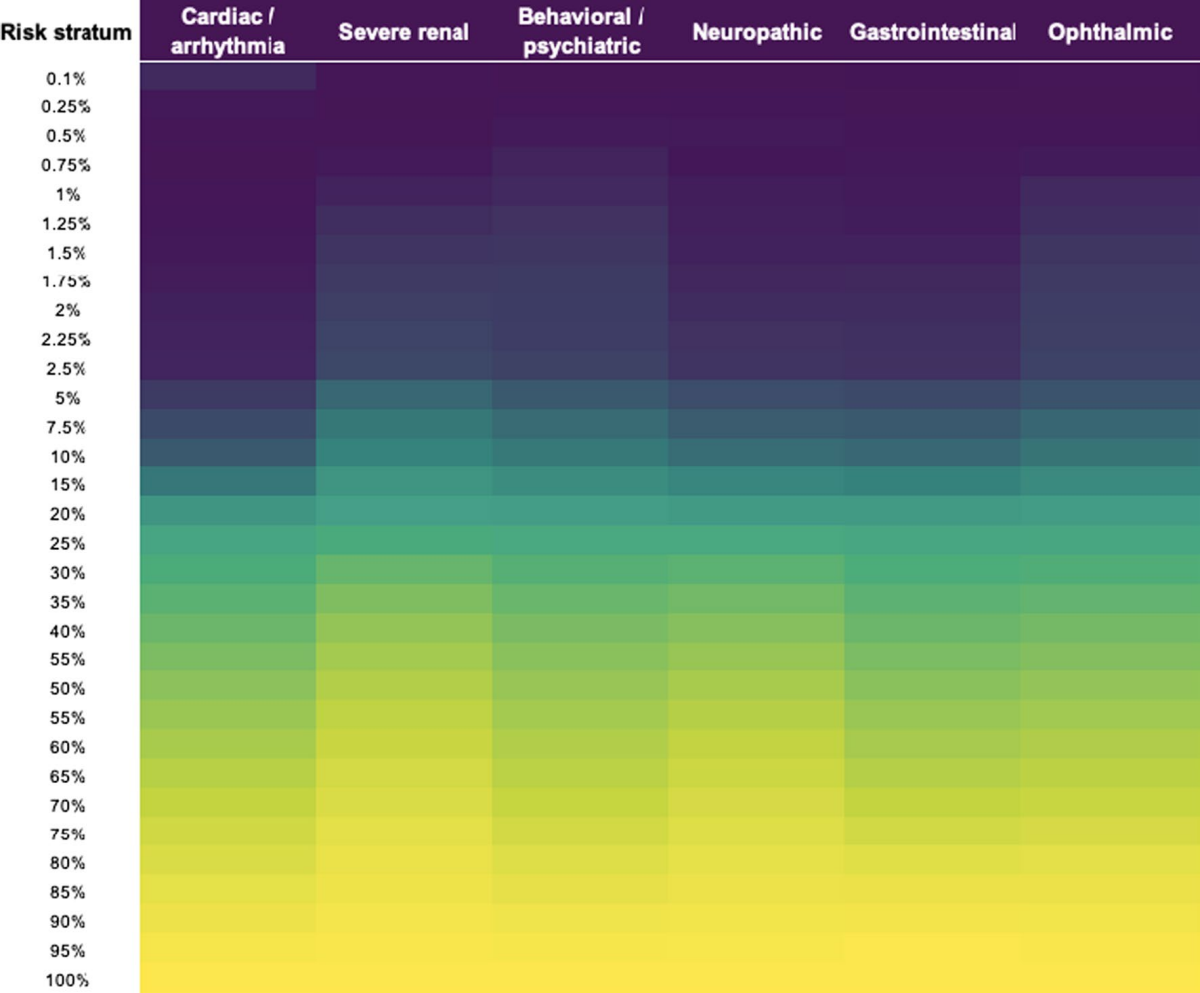
Selected phenotypic features and relative prevalence (portion of patients with evidence of feature) in risk strata, defined following rank-ordering of patients by predicted Fabry disease risk. Darker coloring indicates these features’ increased prevalence in correspondence with increasing risk of Fabry disease. This set of features is a small sample of the hundreds of signals drawn from available data that drove the tool’s analytic performance

Many phenotypic signals contributed to the tool’s overall performance. These signals all displayed differences in frequency of occurrence between patients across risk strata; in general, patients in higher-risk strata had higher rates of occurrence of these phenotypic signals. These differences were quantified by calculating signal prevalence in specific higher-risk strata. Figure 3 displays these calculations for several representative phenotypic signals corresponding to clinically meaningful aspects of Fabry disease presentation. These provide a sample of the broader group of signals contributing to the algorithm’s predictive performance.

Sensitivity analysis examining the tool’s predictive performance in the test set after stratification by sex demonstrated strong analytic results for both men and women. Performance was slightly stronger in the male-only cohort relative to the female-only cohort, but amplification remained substantial within higher-risk cohorts for both groups relative to the overall study population. Amplification within the riskiest 1% subgroup is shown in Table 3.

**Table 3:** Amplification of Fabry disease occurrence in riskiest 1% stratum of the testing set, grouped by sex

Patient subcohort	Amplification relative to assumed 1 in 50,000 prevalence	Projected prevalence
All patients	23.9×	1 in 2090
Male	26.8×	1 in 1867
Female	21.8×	1 in 2291

Performance in the test set also remained very strong within male and female subsets, as illustrated by the respective ROC curves. The AUC for the male-only subcohort was 0.83; for the female-only subcohort, it was 0.82, reflecting balanced performance.

## Discussion

Using a predictive model-based AI approach to pre-screen potential undiagnosed patients may present a more efficient way to focus expensive diagnostic screening efforts on patients at greatest risk of Fabry disease. The AI tool analyzed in this study demonstrated strong overall performance in identifying Fabry disease patients at the individual level using structured medical record data for calibration and prediction. Fabry disease prevalence in higher-risk strata identified by the algorithm was substantially greater than in the background population. This performance was achieved using existing structured real-world health data; no additional Fabry disease-specific data were gathered, nor was information from unstructured data (i.e., clinical notes) used. Even though the data analyzed were de-identified, the AI tool’s ability to recognize relevant phenotypic patterns in individual patients’ histories preserves its potential for patient-specific real-world application. Future applications to identified patient populations will face challenges around consent and data privacy not addressed in this current study, and overcoming these challenges will be crucial to identifying individual patients so proper treatment can be pursued.

The phenotypic signals driving the platform’s predictions of Fabry disease risk correspond very well to clinical knowledge about Fabry disease and its presentation, including both ‘classic’ and late-onset phenotypes. The tool isolated differences in occurrence of phenotypic signals associated with the clearest clinical presentation of advanced disease—specifically, severe renal damage, cardiac arrhythmias, and neuropathies—with more frequent occurrence among those patients determined to be at greatest risk of Fabry disease by the tool. At the same time, the algorithm captured and utilized many more subtle statistical signals as well, including phenotypic signals associated with behavioral health, cerebrovascular damage, and auditory and balance-related symptoms. This abundance of phenotypic signals provided the tool with a rich set of contributing factors to predict Fabry disease risk, reflecting the diversity of patient experience without relying on a limited set of disease characteristics. The authors intend to explore these phenotypic signals at greater length in a subsequent publication.

This identification by the tool of phenotypic signals confirmed by the group of Fabry disease experts to be associated with Fabry disease is strong evidence that the algorithm is operating in concordance with medical, biological, and epidemiological knowledge about the condition. AI models do not always agree with clinical or scientific knowledge in this way and may generate predictions without obvious explanations. This ‘black box’ problem, where AI technology makes predictions that cannot be linked to expert knowledge, does not characterize the tool examined in this study. On the contrary, the algorithm elicited and relies on characteristics known to be associated with Fabry disease. This quality makes the platform an especially promising candidate for real-world clinical application.

This study has several limitations. First, real-world health history data were used for analysis. These data may suffer from missingness or incomplete capture, in addition to errors resulting from mistakes in data recording or transcription. However, this limitation is partly mitigated by the size of the cohort analyzed. The nearly 5000 patients in the confirmed Fabry disease patient group is substantially larger than Fabry disease cohorts found in much of the literature published in this disease area; to the authors’ knowledge, this is one of the largest known Fabry disease patient cohorts studied to date [24, 25].

Second, the training cohort used to calibrate the AI algorithm relied on patients labeled as having Fabry disease—that is, those diagnosed with the disease, or with evidence of a disease-specific treatment in their health history record. Because Fabry disease diagnosis in general does not necessarily correspond to true prevalence, bias may exist within the confirmed Fabry disease cohort. Somewhat surprisingly, the cohort is relatively balanced between male and female patients. The explanation for this balance is not immediately obvious. Diagnosis bias towards ‘classic’ Fabry disease could result in overrepresentation of male patients. However, since female patients experience later onset and longer lifespan than male patients overall [4, 26], survivorship bias could have contributed to greater female representation. Further research on the gender balance in Fabry disease epidemiology is necessary to better contextualize and address these questions. It is important to note that the AI tool in this study draws statistical information from all available aspects of Fabry disease patients’ health histories, including late-onset patterns from dominantly ‘classic’ patients, and can use information in male patients’ histories to inform predictions for females (and vice-versa). Pediatric patients were not analyzed in this study due to data limitations, but future research in pediatric populations may provide additional clarity around patterns in disease manifestation as detected and utilized for AI-driven patient identification.

Finally, the projected prevalence results presented herein assume a baseline population Fabry disease prevalence of 1 in 50,000. This assumption aligns with existing literature around Fabry disease [5] but is likely an underestimate of true prevalence due to underdiagnosis, and is reflective of classic Fabry disease rather than late onset variants. Consequently, projected prevalence estimates in higher-risk strata identified by the tool are likely conservative. If true prevalence is greater than 1 in 50,000, estimates of prevalence in higher-risk groups identified would increase as well.

This study also has many strengths. We evaluated a novel AI tool for identification of undiagnosed Fabry disease patients. The algorithm demonstrated strong analytic performance in identifying patients with Fabry disease, achieving an out-of-sample AUROC of 0.82. In the 1% of patients labeled by the platform as at greatest risk, Fabry disease prevalence was nearly 24-fold greater than in the population overall. The phenotypic characteristics of tool-identified patients correspond to existing literature, represent the multisystemic nature of the disease, and were clinically validated by a group of Fabry experts. These signals’ range covers the full severity spectrum of the disease. This diversity of phenotypic signals provides robustness to the algorithm’s predictive power, which it maintained when tested separately in all-male and all-female subcohorts.

Fabry disease patients continue to face long, difficult journeys from initial presentation of symptoms to eventual diagnosis. These challenges burden Fabry disease patients with clinical manifestations outside the ‘classic’ presentation of the disease, including women, for whom barriers to accurate diagnosis can be especially high. AI technology offers a promising opportunity for earlier diagnosis of Fabry disease by drawing on statistical patterns in from large datasets of patients known to have the condition. Earlier diagnosis, in turn, could result in earlier monitoring, treatment if needed, slowed progression, and better outcomes for patients.

## Conclusions

This study demonstrated that use of a novel AI tool may lead to improved identification of patients with undiagnosed Fabry disease. By labeling patients at disproportionate risk of having the condition, using existing medical record data, the AI tool tested may substantially improve the efficiency of more determinative approaches to Fabry disease diagnosis while continuing to generate new insights into patient characteristics. Future research will focus on clinical implementations of this technology to examine its performance in real-world settings.

## Data Availability

The data that support the findings of this study are available from OM1, Inc. but restrictions apply to the availability of these data, which were used under license for the current study, and so are not publicly available. Data are however available from the authors upon reasonable request and with permission of OM1, Inc.
